# Supplementary material for: Improved Detection of Antibodies against SARS-CoV-2 by Microsphere-Based Antibody Assay
Source: Int J Mol Sci. 2020 Sep 9;21(18):6595. doi: 10.3390/ijms21186595 (PMC7555114; doi:10.3390/ijms21186595)
Supplement: Supplementary file 1 [file ijms-21-06595-s001.pdf]

Supplementary Materials

Supplementary Methods

Cloning and purification of (His)6-tagged recombinant receptor binding domain (RBD) and nucleocapsid protein (NP) of SARS-CoV-2

Cloning and purification of SARS-CoV-2 NP and spike RBD were performed as described previously [1]. Briefly, the genes encoding the spike RBD (amino acid residues 306 to 543 of the spike protein) and full length NP of SARS-CoV-2 were codon-optimized, synthesized, and cloned into the *NdeI* site and *XhoI* site of expression vector pET-28b(+) (Novagen, Madison, Wisconsin, USA) in the respective frame and upstream of the series of six histidine residues. The recombinant RBD and NP were expressed and purified using the $Ni2^{+}$-loaded HiTrap Chelating System (GE Healthcare, Chalfont St Giles, Buckinghamshire, UK) according to the manufacturer's instructions. The purity of RBD and NP was assessed by sodium dodecyl sulfate–polyacrylamide gels (SDS-PAGE) and Western blotting.

Nucleoprotein of severe fever with thrombocytopenia syndrome virus was used as a positive control [2]. The purified RBD and NP were separated electrophoretically in a 12% gel and transferred to a nitrocellulose membrane. After blocking with 3% BSA and 7% skim milk (Sigma-Aldrich, St. Louis, MO, USA), the membrane was incubated with anti-His monoclonal antibodies at a dilution of 1:4000 (Abcam, Cambridge, MA, USA) for 1 hour at room temperature. After washing, the membrane was incubated with 1:4000 diluted goat anti-mouse horseradish peroxidase (Sigma-Aldrich, St. Louis, MO, USA) for 30 mins at room temperature and developed by incubation with the Amersham ECL Advance Western Blotting Detection Kit (GE Healthcare, Chalfont St Giles, Buckinghamshire, UK). The concentration of purified RBD and NP were determined using the Bicinchoninic Acid Protein Assay Kit (Sigma-Aldrich, St. Louis, MO, USA) in accordance with the manufacturer's instructions. Nucleoprotein of severe fever with thrombocytopenia syndrome virus was used as a positive control [2].

Biotinylation of proteins

Purified NP and spike protein RBD was diluted in phosphate-buffered saline (PBS) to 2 mg/mL and dispensed into glass tubes on ice. A total of 2.2 mg of EZ-link™ Sulfo-NHS-Biotin (ThermoFisher Scientific, MA, USA) was dissolved in 0.5 ml sterile H_2O after equilibrating to room temperature. Then, 30 µL of biotin was added to 1 mL of diluted recombinant protein and was kept on ice for 2 h. Biotinylated recombinant proteins were dialyzed using Slide-A-Lyzer™ Dialysis Cassettes (20 Kd for NP and 10 Kd RBD) (ThermoFisher Scientific, Waltham, MA, USA) to remove unbound biotin. The dialyzed proteins were centrifuged at 12,000 rpm for 10 min under 4 °C to eliminate denatured protein. The protein was stored with 50% glycerol under –80 °C. The quantity of biotinylated proteins was determined using Bradford assay. The quantity of biotinylated proteins was determined using Bradford assay and a typical recombinant protein concentration was between 2000 and 3000 µg/mL for NP and 1000-2000 µg/mL for RBD of spike protein (Data not shown).

Enzyme immunoassay for NP and spike RBD

Enzyme immunoassay (EIA) for NP and RBD was performed as described previously [1]. Briefly, 96-well immunoplates (Nunc Immuno modules; Nunc, Denmark) were coated with 100 µL/well (0.1 µg/well) of SARS-CoV-2 NP or spike RBD in 0.05 M NaHCO3 (pH 9.6) overnight at 4 °C and then followed by incubation with a blocking reagent. After blocking, 100 µL heat-inactivated serum samples at 1:100 dilution was added to the wells and incubated at room temperature for 1 h. The attached antibodies were detected using horseradish-peroxidase (HRP)-conjugated goat anti-human IgG antibody (Thermo Fisher Scientific, Waltham, MA, USA). The reaction was developed by adding diluted 3,3′,5,5′-tetramethylbenzidine (TMB) single solution and stopped with 0.3 N

sulfuric acid (H_2SO_4). The optical density (OD) was read at 450–620 nm. A single positive sample was included in each run as a positive control. An archived anonymous sample from 2018 used in our previous study was used as a negative control [9]. To determine the cutoff value for positivity, the mean value of 290 anonymous archived serum specimens from 2018 plus 3 standard deviations was used as the cutoff. The cutoff values are: anti-NP IgG, 0.58; anti-NP anti-RBD IgG 0.54.

Supplementary Tables

Supplementary Table S1. Number of anonymous archived serum specimens in each age group.

Age group (years)	2018 Apr-Jul
0-9	32
10-19	32
20-29	32
30-39	32
40-49	32
50-59	32
60-69	32
70-79	32
80 or above	38
Total	294

Supplementary Figures

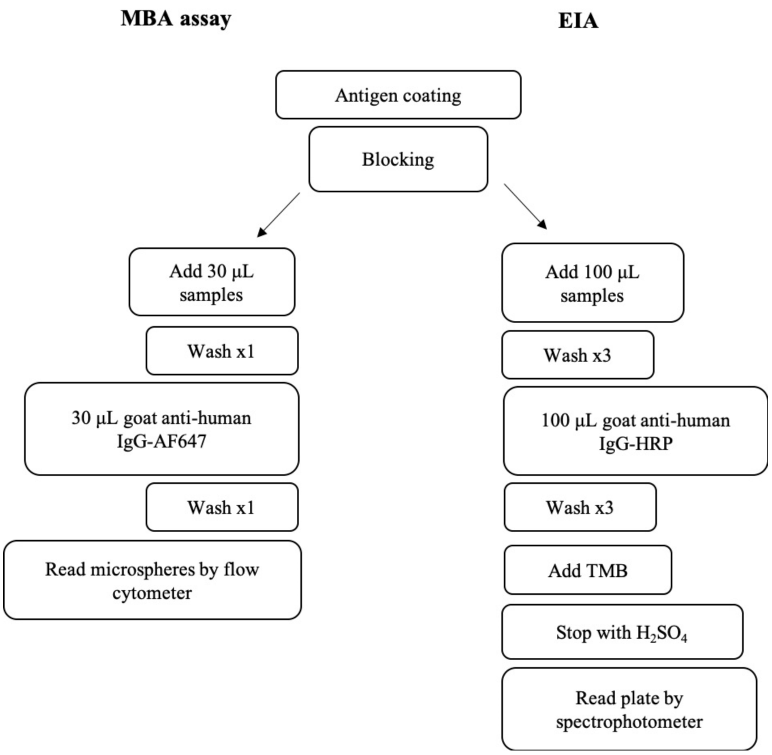

Supplementary Figure S1. Flow chart of different steps in microsphere-based antibody assay (MBA) assay and enzyme immunoassay (EIA).

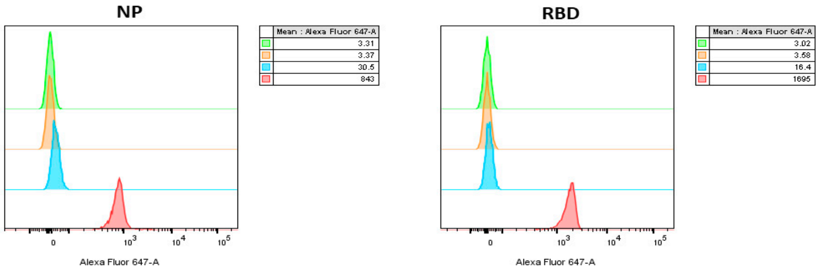

Supplementary Figure S2. Validation of MBA assay with negative and positive controls. SuperAvidin™ coated microspheres were coated with biotinylated nucleocapsid protein (NP) or receptor binding domain (RBD) overnight at 4 °C and blocked with fetal bovine serum (FBS). After 1 h blocking, FBS was removed by centrifugation and serum or diluent (1% BSA) were added to the microspheres and incubated for 2 h at room temperature. Then, microspheres were washed once and goat anti-human IgG-AF647 was added. After 1 h incubation at room temperature, microspheres were washed with phosphate-buffered saline (PBS) and 1% BSA, and the microspheres were ready for flow cytometric analysis. The negative controls include a microsphere without protein coating (green), no serum (diluent only) (orange), and a serum specimen collected in 2018 (blue). The positive control was a serum specimen from a patient with COVID-19 (red).

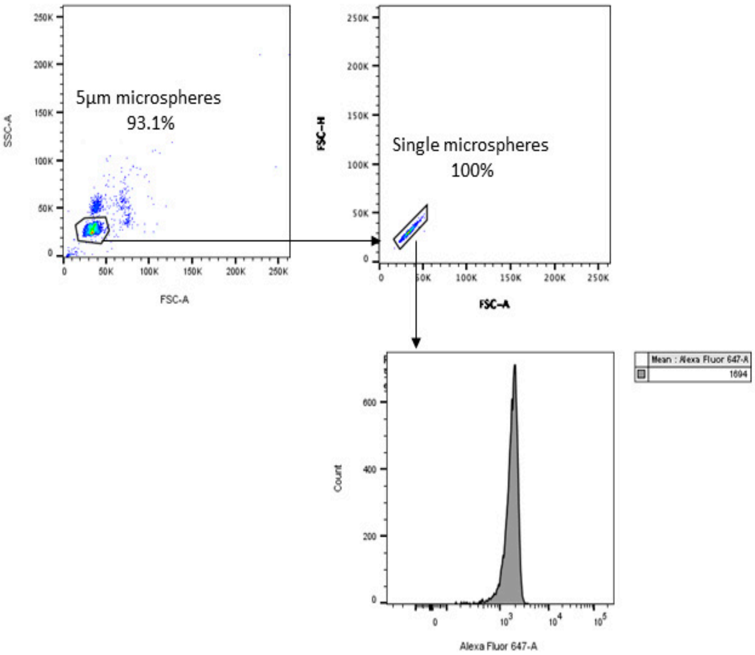

Supplementary Figure S3. Representative flow cytometry images of the analysis of anti-NP or anti-RBD IgG.

References

1. To, K.K.; Tsang, O.T.; Leung, W.S.; Tam, A.R.; Wu, T.C.; Lung, D.C.; Yip, C.C.; Cai, J.P.; Chan, J.M.; Chik, T.S., et al. Temporal profiles of viral load in posterior oropharyngeal saliva samples and serum antibody responses during infection by SARS-CoV-2: an observational cohort study. *The Lancet. Infectious diseases* **2020**, *20*, 565–574, doi:10.1016/s1473-3099(20)30196-1.
2. Yuan, S.; Chan, J.F.; Ye, Z.W.; Wen, L.; Tsang, T.G.; Cao, J.; Huang, J.; Chan, C.C.; Chik, K.K.; Choi, G.K., et al. Screening of an FDA-Approved Drug Library with a Two-Tier System Identifies an Entry Inhibitor of Severe Fever with Thrombocytopenia Syndrome Virus. *Viruses* **2019**, *11*, doi:10.3390/v11040385.
